# A commercialized dietary supplement alleviates joint pain in community adults: a double-blind, placebo-controlled community trial

**DOI:** 10.1186/1475-2891-12-154

**Published:** 2013-11-25

**Authors:** David C Nieman, R Andrew Shanely, Beibei Luo, Dustin Dew, Mary Pat Meaney, Wei Sha

**Affiliations:** 1 Appalachian State University, Human Performance Lab, North Carolina Research Campus, 600 Laureate Way, Kannapolis, NC 28081, USA; 2 Key Laboratory of Exercise and Health Sciences of Ministry of Education, Shanghai University of Sport, Shanghai, China; 3 Bioinformatics Services Division, University of North Carolina at Charlotte, North Carolina Research Campus, Kannapolis, NC 28081, USA

**Keywords:** Glucosamine, Inflammation, Quality of life, Physical function

## Abstract

**Background:**

The purpose of this study was to assess the effect of 8-weeks ingestion of a commercialized joint pain dietary supplement (InstaflexTM Joint Support, Direct Digital, Charlotte, NC) compared to placebo on joint pain, stiffness, and function in adults with self-reported joint pain. InstaflexTM is a joint pain supplement containing glucosamine sulfate, methylsufonlylmethane (MSM), white willow bark extract (15% salicin), ginger root concentrate, boswella serrata extract (65% boswellic acid), turmeric root extract, cayenne, and hyaluronic acid.

**Methods:**

Subjects included 100 men and women, ages 50-75 years, with a history (>3 months) of joint pain, and were randomized to Instaflex™ or placebo (3 colored gel capsules per day for 8 weeks, double-blind administration). Subjects agreed to avoid the use of non-steroidal anti-inflammatory drugs (NSAID) and all other medications and supplements targeted for joint pain. Primary outcome measures were obtained pre- and post-study and included joint pain severity, stiffness, and function (Western Ontario and McMaster Universities [WOMAC]), and secondary outcome measures included health-related quality of life (Short Form 36 or SF-36), systemic inflammation (serum C-reactive protein and 9 plasma cytokines), and physical function (6-minute walk test). Joint pain symptom severity was assessed bi-weekly using a 12-point Likert visual scale (12-VS).

**Results:**

Joint pain severity was significantly reduced in Instaflex™ compared to placebo (8-week WOMAC, ↓37% versus ↓16%, respectively, interaction effect P = 0.025), with group differences using the 12-VS emerging by week 4 of the study (interaction effect, P = 0.0125). Improvements in ability to perform daily activities and stiffness scores in Instaflex™ compared to placebo were most evident for the 74% of subjects reporting knee pain (8-week WOMAC function score, ↓39% versus ↓14%, respectively, interaction effect P = 0.027; stiffness score, ↓30% versus ↓12%, respectively, interaction effect P = 0.081). Patterns of change in SF-36, systemic inflammation biomarkers, and the 6-minute walk test did not differ significantly between groups during the 8-week study

**Conclusions:**

Results from this randomized, double blind, placebo-controlled community trial support the use of the Instaflex™ dietary supplement in alleviating joint pain severity in middle-aged and older adults, with mitigation of difficulty performing daily activities most apparent in subjects with knee pain.

**Trial registration:**

ClinicalTrials.gov Identifier: NCT01956500

## Background

Joint pain is reported by 32% of U.S. adults, and increases with age reaching 50% prevalence among the elderly [1]. Joint pain is slightly more prevalent among women (33%) than men (31%), and among white, non-Hispanic adults (33%), than black, non-Hispanic adults (32%) and Hispanic adults (25%). The knee is the most common site of joint pain regardless of age or gender. Joint pain is associated with substantial activity limitation, work disability, and reduced quality of life. Adults with joint pain are more likely to report arthritis-attributable activity limitations, fair or poor health, inability to work, low sleep duration (<6 hours per day), and psychological distress. Predictors of knee pain include older age, weight gain and obesity, and previous knee injury, with the combination of weight loss with exercise a well-recognized intervention to alleviate symptoms and improve function [2,3].

Conventional treatment of joint pain with non-steroidal anti-inflammatory drugs (NSAIDs) and other analgesics is associated with gastrointestinal and cardiovascular side effects, and other adverse health effects [4]. Use of alternative supplements is reported by 47% of individuals with knee osteoarthritis, and well-designed human trials are needed to identify effective analgesic alternatives [5]. The most widely used and studied joint pain supplements include those related to chondroprotection such as glucosamine, chondroitin, methylsulfonylmethane, collagen hydrolysates, and hyaluronic acid [6-8]. Herbal and neutraceutical products have been investigated including avocado-soybean unsaponifiables, curcumin and turmeric extract, epigallocatechin gallate and green tea extract, resveratrol, nobiletin and citrus fruits, omega-3 fatty acids, cat’s claw, Boswella serrata, white willow bark, antioxidants, ginger, cayenne, barberry, hu zhang, oregano, rosemary, Baikal skullcap, Chinese goldthread, Indian holy basil, and many others [6,9-11].

Inflammation is a prominent mechanism leading to cartilage degeneration, and there is increasing realization that therapy must involve both cartilage protection and anti-inflammatory actions [7]. The Instaflex™ Joint Support supplement (Instaflex) (Direct Digital, Charlotte, NC) features a cocktail of eight chondroprotective and anti-inflammatory ingredients that may relieve joint pain discomfort and improve function. Ingredients include glucosamine sulfate [12], methylsufonlylmethane [13-15], white willow bark extract [16], ginger root concentrate [17,18], Boswella serrata extract [19,20], turmeric root extract [21], cayenne [22], and hyaluronic acid [23]. Each of these ingredients has been studied separately providing some scientific support for alleviation of joint pain, but the composite product has not yet been tested in humans using a randomized, double-blinded, placebo-controlled research design.

The primary purpose of this study was to assess the effect of 8-weeks ingestion of Instaflex compared to placebo on joint pain, stiffness, and function utilizing validated questionnaires and the 6-minute walk test. Secondary outcomes for this trial included the measurement of systemic inflammation biomarkers.

## Methods

### Subjects

Subjects were recruited through mass advertising during a one-month period, and included 108 men and women, ages 50–75 years. Inclusion and exclusion criteria for this study included:

1. Self-reported history (>3 months) of joint pain in the knees, hip, ankles, shoulders, or hands. Minimum symptom severity was ensured by using a WOMAC pain index score of at least 2 points.

2. No history of regular NSAID use (e.g., ibuprofen, aspirin) during the previous two weeks, and willingness to avoid NSAIDs use during the 8-week study.

3. Not on other medications (e.g., analgesic gels, arthritis medications, other anti-inflammatory drugs) or supplements (in particular, glucosamine and chondroitin) for joint pain for the previous two weeks, and willing to avoid use of these during the 8-week study.

4. No serious medical problems (current cancer case, severe rheumatoid arthritis, recent heart attack, recent stroke, congestive heart failure, ulcers, kidney disease, or other disease that would interfere with study participation).

5. No psychiatric disorder or other condition that might interfere with self-assessment ability.

6. Willingness to follow all study procedures including randomization to one of two groups, and to stay weight stable during the study.

7. Able to walk for at least 6 min at a moderate-to-brisk pace.

8. No history of allergic reactions to shellfish products or products containing aspirin.

Written informed consent was obtained from each subject, and the experimental procedures were approved by the institutional review board for human studies at Appalachian State University. Paracetamol (i.e., acetaminophen as found in Tylenol) was allowed as a rescue medicine for pain during the study as needed, with usage recorded.

### Research design

This study employed a placebo-controlled, randomized, double-blind design with two groups. Subjects were recruited from one site in the Charlotte, NC, metropolitan area, and randomized using a stratified block design to ensure similar numbers of males and females to one of two groups: Instaflex™ Joint Support (Instaflex) or placebo. Supplements were administered in double-blind fashion during an 8-week period. Blood samples were drawn from an antecubital vein with subjects in the seated position pre- and post-study in the late afternoon. During the pre-study lab visit, subjects reviewed and signed the consent form, completed a medical-health questionnaire to verify medical history and lifestyle habits, completed a 2-week retrospective symptom log, and were measured for height and weight. Height was measured using a stadiometer, and body weight measured using a digital scale (Tanita Corporation of America, Inc., Arlington Heights, IL). Subjects completed the Western Ontario and McMaster Universities (WOMAC) and health-related quality of life questionnaires (Short Form 36 or SF-36). Subjects then engaged in the 6-minute walk test on an indoor track. Finally, each subject received a supplement organizer tray with 8-weeks supply and written instructions for taking the supplements. These outcome measures were repeated during the second lab visit.

### Instaflex joint support supplement

Supplements (Instaflex, placebo) were prepared in colored gel capsules (3 per day, identical looking), and given to the subjects in supplement organizer trays. Subjects ingested 3 capsules/day: one capsule each in the morning, at noontime, and in the evening. The placebo capsules contained magnesium stearate, an inert substance. Compliance was monitored with bi-weekly email messages, and by counting unused capsules when subjects returned the supplement trays at the end of the 8-week study. If subjects missed one, two, or three days of taking supplements, subjects were asked to double up usage until back on schedule. Subjects missing more than three days of taking the supplements were asked to leave the study. The Instaflex supplement contained the following ingredients (in 3 capsules): Glucosamine sulfate (1500 mg), methylsufonlylmethane (MSM) (500 mg), white willow bark extract (standardized to 15% salicin) (250 mg), ginger root concentrate (50 mg), boswella serrata extract (standardized to 65% boswellic acid) (125 mg), turmeric root extract (50 mg), cayenne 40 m H.U. (50 mg), and hyaluronic acid (4.0 mg).

### WOMAC questionnaire

The WOMAC™ Index is a tri-dimensional self-administered questionnaire to assess joint pain (five items), stiffness (two items), and physical function (16 items), and can be completed in less than 5 minutes [24]. The Likert version was used in this study, with five response levels for each item, representing different degrees of intensity (none, mild, moderate, severe, or extreme) that were scored from 0 to 4. Scores for the WOMAC were determined by adding items scores within each index (pain, stiffness, and function), and an aggregate score by adding scores from the three indexes. The worst possible severity scores for pain, stiffness, function (limitation of physical function), and total were 20, 8, 64, and 92 points, respectively.

### SF-36 questionnaire

The SF-36 is a generic health-related quality of life instrument [25]. The 36 items in the SF-36 cover eight domains (physical functioning, limitations due to physical health, bodily pain, general health, vitality, social functioning, and limitations related to emotional and mental health) and the physical and mental summary scales. The scores for the SF-36 scales range from 0 to 100, with a higher score indicating better health status.

### 2-week retrospective symptom logs and 12-point likert visual scale (12-VS) for joint pain

The retrospective symptom logs were completed every two weeks during the study, and included questions on digestive health (constipation, heartburn, bloating, diarrhea, and nausea), hunger levels (morning, afternoon, and evening), energy levels (morning, afternoon, and evening), sickness (fever, cough, sore throat, stuffy nose, runny nose, and headache), pain (joint, muscle, and back), allergies, stress level, focus/concentration, and overall well-being [26]. For each of these including joint pain (12-VS), subjects were asked to place an “X” in one of 12 boxes (labeled 1 to 12) lined up in one row that best matched the symptoms or feelings experienced during the past two weeks, with 1 = none at all, 3 = low, 6 = moderate, 9 = high, and 12 = very high levels of the symptom or feeling.

### 6-minute walk test

The 6-minute walk test is commonly used to assess whether or not an intervention is associated with improved ability to walk faster during a 6-minute period in individuals with various health problems [27]. Subjects dressed in comfortable clothing and shoes, and were instructed to walk as far as possible in 6 minutes on an indoor track while receiving encouragement from the staff. A practice 3-min walk test was conducted prior to recording measurements during the 6-minute walk test.

### Blood measures

Serum C-reactive protein (CRP) and a serum comprehensive diagnostic chemistry panel were analyzed by the clinical hematology laboratory at the Carolina Medical Center (Charlotte, NC). CRP was measured using an LX-20 clinical analyzer (Beckman Coulter Electronics, Brea, CA). Total plasma concentrations of four inflammatory cytokines (interleukin-6 [IL-6], tumor necrosis factor alpha [TNFα], IL-8, and IL-10) were determined using an electrochemiluminescence based solid-phase sandwich immunoassay (Meso Scale Discovery, Gaithersburg, MD). All samples and provided standards were analyzed in duplicate, and the intra-assay coefficient of variation (CV) ranged from 1.7% to 7.5%, and the interassay CV ranged from 2.4% to 9.6%, for all cytokines.

### Statistical procedures

Data analysis was performed for the 100 subjects successfully completing the study. Data are expressed as mean ± SE. Subject characteristics were compared using independent Student’s *t* tests between the treatment (Instaflex) and placebo groups (Table 1). The blood biomarker data was analyzed by 2 (groups) × 2 (time points) repeated measures ANOVA, where blood biomarker level (continuous variable) was the response variable; group, time, and group × time interaction effect were predictor variables. Each biomarker was analyzed separately. The composite scores from questionnaires were also analyzed by 2 × 2 repeated measures ANOVA. Individual answers in questionnaires and symptom log were analyzed by generalized estimating equations, where each individual question (categorical variable) was the response variable; group, time, and group × time (2 × 2 for questionnaires, and 2 × 5 for symptom log) interaction effect were predictor variables. Each individual question was analyzed separately. For both repeated measures ANOVA and generalized estimating equation, response variables with significant group × time interaction effect were considered to be significantly affected by the Instaflex supplement, and the interaction P-value was used for the group contrast. For symptom log variables with five time points, P-values at each time point represent independent student’s *t*-test contrasts between groups for changes from pre-study. All analyses were performed in SAS (version 9.2, SAS Institute, Inc., Cary, NC).

**Table 1:** Subject characteristics and pre- and post-study (8 weeks) body mass and BMI (N = 100) (mean ± SE)

**Variable**	**Instaflex (N = 49; 41 F, 8 M)**	**Placebo (N = 51; 42 F, 9 M)**	**Range**	**P-Value ( *t *-test or ANOVA)**
Age (yrs)	57.6 ± 0.9	58.3 ± 0.8	50-75	0.554
Height (m)	1.67 ± 0.01	1.65 ± 0.01	1.46-1.84	0.106
Body mass (kg)				
Pre-Study	89.3 ± 2.6	84.8 ± 2.3	54.5-140.9	0.422
Post-Study	89.4 ± 2.6	84.7 ± 2.3		
BMI				
Pre-Study	31.6 ± 0.9	30.9 ± 0.8	21.1-48.0	0.536
Post-Study	31.6 ± 0.9	30.8 ± 0.8		

## Results

Of the 108 subjects recruited into the study, 101 (N = 50 Instaflex and N = 51 placebo) completed all aspects of the study. Subjects dropping out of the study (N = 4 from Instaflex, N = 3 from placebo) cited health issues (N = 4) or noncompliance with the supplementation regimen (N = 3). One subject (Instaflex) was removed the data analysis because rescue medication use reached daily levels by the end of the study. All other subjects complied fully with study requirements and the supplementation protocol, and no unused capsules were left in the supplement trays. Subject characteristics and pre- and post-study body mass and BMI values are compared between groups in Table 1, with no significant group differences measured. Of the 100 subjects completing the study, 55% of the subjects had a BMI ≥ 30 kg/m^2^ (obese), and 30% a BMI of 25–29.9 kg/m^2^ (overweight). Subjects were predominately female, with eight males in the Instaflex group and 9 males in the placebo group (gender Chi-square P-value = 0.861). For all subjects combined, 74.2% reported knee pain, 55.7% upper limb joint pain, 41.2% hip pain, 24.7% lower limb joint pain, and 18.6% back pain. Thirty-one percent of subjects reported arthritis, but this was not confirmed through physician diagnosis. Chi-square analysis revealed no group differences for these percentages.

**Figure 1:**
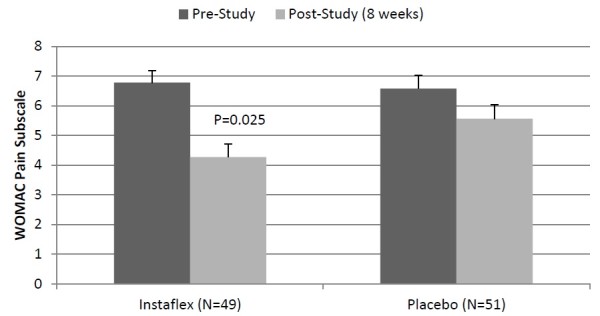
**WOMAC joint pain in Instaflex compared to placebo (interaction effect, P = 0.025).** The P-value in the graph represents the pre-to-post study contrast between Instaflex and placebo groups.

The pattern of change in the total WOMAC score tended to be different between groups (Instaflex 29.4 ± 2.0 to 19.0 ± 1.9, placebo 30.0 ± 2.0 to 24.6 ± 1.0, interaction effect P = 0.074). Joint pain severity was significantly reduced in Instaflex compared to placebo (8-week WOMAC, ↓37% versus ↓16%, respectively, interaction effect P = 0.025) (Figure 1). Interaction effects for the WOMAC joint stiffness (↓26% versus ↓18%, respectively, interaction effect P = 0.325) and joint function index scores (↓36% versus ↓19%, respectively, interaction effect P = 0.117) were not significant.

**Figure 2:**
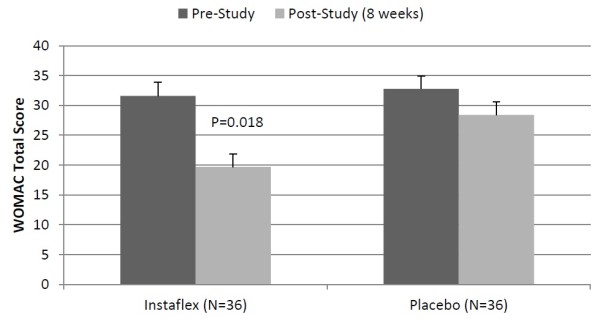
**WOMAC total scores in subjects reporting knee pain (interaction effect, P = 0.018).** The P-value in the graph represents the pre-to-post study contrast between Instaflex and placebo groups.

**Figure 3:**
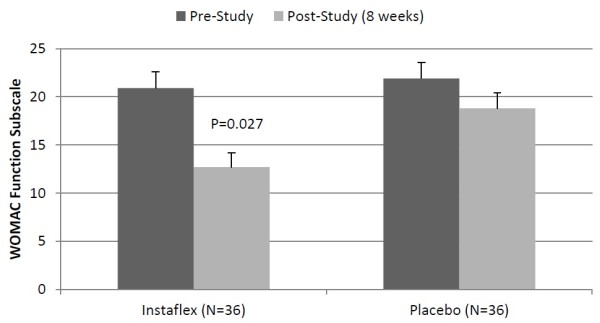
**WOMAC function scores in subjects reporting knee pain (interaction effect, P = 0.027).** The P-value in the graph represents the pre-to-post study contrast between Instaflex and placebo groups.

In a separate analysis of the 74% of subjects reporting knee pain (73% in Instaflex, 75% in placebo), decreases in WOMAC total and pain, stiffness, and function index scores were greater for Instaflex compared to placebo [8-week WOMAC total score, ↓38% versus ↓17%, respectively, interaction effect P = 0.018 (Figure 2); pain score, ↓39% versus ↓11%, respectively, interaction effect P = 0.014; stiffness score, ↓30% versus ↓12%, respectively, P = 0.081; function score, ↓39% versus ↓14%, respectively, P = 0.027] (Figure 3).

**Figure 4:**
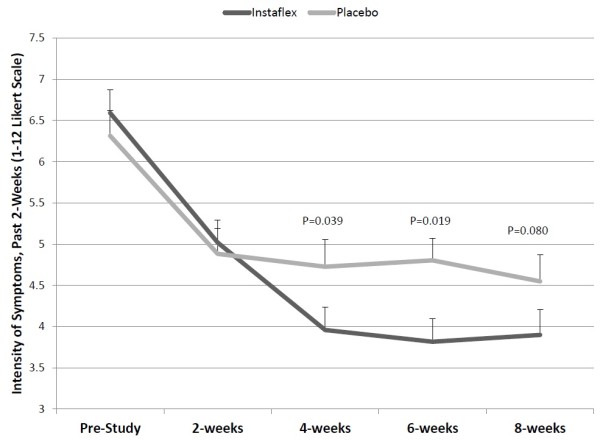
**Joint pain symptom severity assessed bi-weekly using the 12-point Likert visual scale (12-VS) (2 × 5 interaction effect, P = 0.0125).** P-values at each time point represent independent student’s *t*-test contrasts between groups for changes from pre-study.

Joint pain symptom severity assessed bi-weekly using the 12-point Likert visual scale (12-VS) from the symptom logs showed significant reductions for Instaflex compared to placebo starting at week 4 (interaction effect, P = 0.0125) (Figure 4). Other data collected from the 2-week retrospective symptom logs showed no group differences (data not shown).

Patterns of change in responses from the SF-36 (data not shown, total score, interaction effect, P = 0.986), systemic inflammation biomarkers (Table 2), and the 6-minute walk test (Instaflex 561 ± 10.8 to 558 ± 10.7 meters, placebo 558 ± 10.0 to 545 ± 10.7 meters, interaction effect P = 0.233) did not differ significantly between groups during the 8-week study. Diagnostic chemistry panels revealed no group differences over the 8-week study for all liver and kidney function, and metabolic measures including albumin, alkaline phosphatase, alanine aminotransferase, aspartate aminotransferase, blood urea nitrogen, calcium, chloride, carbon dioxide, creatinine, glucose test, potassium, sodium, total bilirubin, and total protein (data not shown).

**Table 2:** Serum C-reactive protein (CRP) and selected plasma cytokine data pre- and post-study (8 weeks) (mean ± SE)

**Variable**	**Instaflex (N = 49)**	**Placebo (N = 51)**	**Interaction effect, P-Value**
CRP (mg/L)			
Pre-Study	4.05 ± 0.64	3.77 ± 0.43	0.518
Post-Study	3.56 ± 0.56	3.50 ± 0.41	
IL-6 (pg/ml)			
Pre-Study	2.12 ± 0.20	1.80 ± 0.20	0.991
Post-Study	2.16 ± 0.17	1.84 ± 0.17	
IL-8 (pg/ml)			
Pre-Study	5.39 ± 0.35	5.55 ± 0.34	0.143
Post-Study	6.41 ± 0.40	7.13 ± 0.39	
TNF-α (pg/ml)			
Pre-Study	4.19 ± 0.16	4.32 ± 0.16	0.380
Post-Study	4.10 ± 0.17	4.32 ± 0.17	
IL-10 (pg/ml)			
Pre-Study	1.24 ± 0.11	1.39 ± 0.10	0.760
Post-Study	1.23 ± 0.08	1.35 ± 0.08	

## Discussion

This 8-week randomized, double-blind, placebo-controlled study with 100 community adults reporting joint pain showed that Instaflex is efficacious and safe to use, with no adverse symptomology or negative effects on general metabolism and liver and kidney function. Instaflex caused significant reductions in joint pain for the whole group. Among the 74% of subjects with knee pain, difficulties performing daily activities were attenuated. Joint pain reduction effects from Instaflex become apparent by the fourth week of supplementation, and occurred without changes in systemic inflammation or distance walked in six minutes.

The primary outcomes of this study were the pain, stiffness, and function indexes of the WOMAC. The magnitude of decrease in joint pain for all subjects in the Instaflex (↓37%) compared to placebo (↓16%) group, and decrease in difficulties performing daily activities for those with knee pain (↓39% vs. ↓14%), is comparable to or higher than what has been reported in other studies using chondroprotective or anti-inflammatory dietary supplements in subjects with osteoarthritis [8,12-14,28-33]. For comparison with a lifestyle change factor, one study of 250 subjects showed that weight loss of 5% and higher (median 11.1 kg) resulted in a 54% decrease in the WOMAC pain index after adjustment for age, sex, and baseline weight [3].

Most previous studies testing the efficacy of dietary supplements on joint pain reduction have compared placebo to one or two of the chondroprotective components including glucosamine (typical dose, 1,000-1,500 mg/day), chondroitin sulfate (800–1,200 mg/day), methylsulfonylmethane (1,500 mg/day), collagen hydrolysates, and hyaluronic acid [6-8]. Results from these studies have been mixed [6,8,12,13,15,23,28] and the most recent meta-analysis of glucosamine and chondroitin was non-supportive [29].

There is increasing evidence that nutraceutical-based combinations of chondroprotective and/or anti-inflammatory components are modestly effective in reducing joint pain within 4–16 weeks without measurable side effects [9,11]. Instaflex (3 capsules daily portion) combines 1500 mg of glucosamine sulfate, 500 mg methylsufonlylmethane, and 4 mg hyaluronic acid with several anti-inflammatory substances including white willow bark extract, ginger root concentrate, boswella serrata extract, turmeric root extract, and cayenne. Other combinations of nutraceuticals, botanicals, and dietary supplements that have shown efficacy in reducing joint pain symptoms in double-blind, placebo-controlled studies include *Phellodendron amurense* bark and *Citrus sinensis* peel extracts standardized to berberine and polymethoxylated flavones [30], lemon verbena extract (14% verbascoside) with fish oil [31], a product containing dried extracts from six Asian herbs [32], and a mixture of glucosamine, chondroitin, and quercetin glycoside [33].

Each of the anti-inflammatory components of Instaflex has been studied separately for influences on joint pain management, but little is known regarding potential synergistic effects when these are combined in smaller amounts than used in single component studies. The active principle in white willow bark extract is salicin, an alcoholic β-glucoside closely related to acetylsalicylic acid in aspirin. The daily dose of Instaflex supplement contains approximately the equivalent of ¼ an aspirin tablet. Studies using larger doses of white willow bark providing about 240 mg/day salicin for periods of up to six weeks generally support modest pain relief [16,34].

More than 100 compounds have been reported from ginger, including gingerols that possess anti-inflammatory, analgesic, and cardiotonic effects [18,35]. Ginger has gained considerable attention in developed countries in recent years, especially for its use in the treatment of inflammatory conditions, and a study of patients with osteoarthritis showed a moderate effect of ginger extract in reducing symptomatology [17]. Boswellia serrata is Indian frankincense or Salai, and has been used for centuries as a treatment for arthritis. Animal studies and pilot clinical trials support the potential of Boswellia serrate gum resin extract in the treatment of a variety of inflammatory diseases including osteoarthritis [19,20,36], and a study of 75 patients with osteoarthritis showed that Boswella serrate extract safely reduced pain and improved physical functioning [20].

Turmeric contains over 300 different components including the active ingredient curcumin (3–5%) [37]. In vitro and animal research shows curcumin is a highly pleiotropic molecule capable of interacting with numerous molecular targets involved in inflammation [21]. Few human trials have been published, but one study of 107 patients with knee osteoarthritis showed that turmeric extract was as efficacious as ibuprofen in alleviating symptoms [38]. Cayenne or red pepper spice contains capsaicin that activates the transient potential receptor vanilloid 1 (TRPV1) channel involved in some aspects of inflammation control [39]. One review of several studies indicates that cayenne compared to placebo exerts modest effects for pain relief [22].

## Conclusions

This 8-week randomized community trial with middle-aged and older adults supports the use of Instaflex in reducing joint pain and improving the ability to perform daily activities, especially in subjects reporting knee pain. Joint pain reduction in the Instaflex compared to placebo group was measurable by the fourth week of the study, indicating a relatively rapid response. Symptom logs and diagnostic chemistries did not reveal any adverse effects associating with the oral ingestion of Instaflex during the 8-week period of this study. Instaflex contains eight chondroprotective and anti-inflammatory ingredients in amounts that are below levels commonly used in other studies evaluating efficacy of single components. Further research should help reveal whether this cocktail of nutraceuticals can be optimized further by changes in relative quantities and ingredients, and underlying mechanisms responsible for the joint pain reduction measured in this study.

## Abbreviations

12-VS: 12-point Likert visual scale; BMI: Body mass index; CRP: C-reactive protein; WOMAC: Western Ontario and McMaster Universities; MSM: Methylsufonlylmethane; NSAID: Non-steroidal anti-inflammatory drugs; SF-36: Short form 36 quality of life questionnaire; TRPV1: Transient potential receptor vanilloid.

## Competing interests

This study was funded by Direct Digital. Direct Digital provided the Instaflex and placebo supplements used in this study. None of the authors report competing interests.

## Authors’ contributions

DCN participated in setting up the research design, coordinated the study and data collection, interpreted results, and drafted the manuscript. RAS participated in setting up the research design, interpreted results, and drafted the manuscript. BL participated in blood sample analysis and drafting of the manuscript. DD participated in setting up the research design, coordinated the study and data collection, interpreted results, and drafted the manuscript. MPM participated in blood sample analysis and drafting of the manuscript. WS participated in statistical analysis and drafted the manuscript. All authors read and approved the final manuscript.
